# Securing Embedded System from Code Reuse Attacks: A Lightweight Scheme with Hardware Assistance

**DOI:** 10.3390/mi14081525

**Published:** 2023-07-29

**Authors:** Zhenliang An, Weike Wang, Wenxin Li, Senyang Li, Dexue Zhang

**Affiliations:** College of Electronic and Information Engineering, Shandong University of Science and Technology, Qingdao 266590, China; anzhenliang@sdust.edu.cn (Z.A.); wenxin_li@sdust.edu.cn (W.L.); lisenyang@sdust.edu.cn (S.L.)

**Keywords:** embedded system, code reuse attack, control flow integrity, hardware-assisted lightweight scheme

## Abstract

The growing prevalence of embedded systems in various applications has raised concerns about their vulnerability to malicious code reuse attacks. Current software-based and hardware-assisted security techniques struggle to detect or block these attacks with minor performance and implementation overhead. To address this issue, this paper presents a lightweight hardware-assisted scheme to enhance the security of embedded systems against code reuse attacks. We develop an on-chip lightweight hardware shadow stack to validate target addresses at runtime for backward-edge control flow integrity, which backs up valid return addresses during function calls and automatically verifies actual return addresses during the return phase. Additionally, we propose a lightweight stream cipher circuit that encrypts and decrypts critical stack data related to control flow manipulation, preventing attackers from analyzing or tampering with them. When designing and implementing the security mechanism for embedded systems, we fully consider the constraints of limited system resources and performance, optimizing both the architecture design and implementation of the proposed hardware. Finally, we integrate both the proposed lightweight hardware shadow stack and the runtime data encryption hardware into the OR1200 processor. We have verified the system security function on the Terasic DE1-SoC FPGA platform and evaluated the system performance as well as implementation overhead. The results show that the proposed lightweight hardware-assisted scheme can provide a dedicated defense capability against code reuse attacks for embedded systems, with an average system performance overhead of 0.39% and an area footprint of 0.316 mm^2^.

## 1. Introduction

With the widespread application of embedded systems in various fields, security issues caused by malicious attacks have become increasingly prominent. The code reuse attack (CRA) leverages existing valid gadgets in a program to construct an attack, tampering with the control flow transfers and hijacking the program execution, which has become one of the most effective methods to compromise the embedded systems. Some representative software-based and hardware-assisted security techniques such as Write XOR eXecution (W^X), address space layout randomization (ASLR), and code integrity checkers, can hardly detect or block the code reuse attacks.

Some researchers have proposed several solutions to defend against code reuse attacks, mainly focused on detecting invalid program behaviors at runtime or preventing attacks before their conduction. Exploiting control flow transfer vulnerability is one of the most direct ways to launch code reuse attacks. The control flow integrity (CFI) checking mechanism is an effective method to detect abnormal control flow transfers. In these methods, the valid transfer paths are analyzed first. Then, the runtime transfer path is monitored, and the alarms will be triggered if any control flow transfer path deviates from the legitimate path [1]. The current mechanisms for control flow integrity checking can be classified as either fine-grained or coarse-grained, depending on the level of granularity in their analysis.

The fine-grained CFI performs static analysis on the code before execution, constructs corresponding control flow graphs, and verifies the validity of all transfer instructions at runtime. Although fine-grained checking mechanisms offer high security, they typically lead to considerable performance overhead [2], which makes their practical application in embedded systems difficult. Hu proposed the unique code target (UCT) CFI attribute and developed an uCFI prototype based on this attribute. This prototype is responsible for checking control flow transfer instructions, recording relevant information, and determining whether the program’s control flow violates the rules based on the runtime-monitored ICT targets. The uCFI model introduced approximately 10% performance overhead [3]. Chen proposed a fine-grained CFI-based detection method to defend against kernel-level code reuse attacks. This model improves the reliability and security of the detection method based on the CFI tag instruction tool and CFI constraint rules. The results showed that this method’s performance overhead is about 60% [4]. Park proposed a random CFI (RCFI) detection mechanism to reduce the performance overhead of fine-grained CFI. RCFI adopts a randomly selective verification strategy, reducing the performance overhead by decreasing the number of indirect branch verifications. However, while RCFI reduces the actual verification quantity, it also leads to more potential detection vulnerabilities and reduced security [5].

Coarse-grained CFI mechanisms primarily rely on inspecting the sensitive metadata of code reuse attacks, resulting in a significant reduction in performance overhead but inadequate security. To defend against stack buffer overflow attacks, Zhang designed a dynamic shadow stack (RS-Stack) to store sensitive data such as return addresses. Randomizing the address of the shadow stack serves as a preventive measure against attackers attempting to locate it. However, this protection mechanism may result in increased performance overhead [6]. Hardware-implemented shadow stacks can store sensitive data, such as return addresses and pointers, but they have high hardware resource overhead and limited capacity. Dang’s software-implemented shadow stack requires additional storage security protection. It needs control flow tracing and stack management during program execution, significantly impacting processor performance, and resulting in a more than 10% overhead [7].

Most of the research has focused on detecting invalid control flow transfers after a CRA attack. Some other researchers have proposed several proactive defense mechanisms against code reuse attacks, such as code pointer integrity [8], encryption-based control flow integrity [9,10], and security-enhanced address space layout randomization [11,12]. Qiu proposed a lightweight encryption architecture based on the Advanced Encryption Standard (LEA-AES) [13]. LEA-AES encrypts and decrypts function return addresses and indirect jump instructions to ensure that the function call and return instructions, as well as indirect jumps, are not exploited by the attackers. Clercq implemented SOFIA, a hardware security architecture, which adopts a fine-grained control flow integrity method based on data encryption [14]. It effectively protects the program from being compromised by code reuse attacks at runtime. However, these two methods’ encryption and decryption operations impose significant runtime overhead on the system due to frequent function calls and returns during program execution. Furthermore, the implementation of these systems is limited in practicality for embedded applications due to the additional hardware resources required.

In this paper, we develop and implement a lightweight hardware-assisted CFI protection scheme to address CRA attacks in embedded systems, while considering their stringent real-time requirements, limited performance capabilities, and prohibitive additional hardware overheads. This mechanism is implemented with a focus on stack data security, primarily involving backward-edge CFI verification utilizing lightweight shadow stacks and CFI protection through sensitive data encryption.

In the design of backward-edge CFI verification, we design a lightweight hardware shadow stack inspired by the coarse-grained CFI to verify the validation of target addresses for backward edges at runtime. This mechanism ensures the secure processing of backward-edge transfers, mainly for returns from functions. Most of the previous research on shadow stacks tried to back up whole stack data and encountered issues such as significant resource and performance overhead. Formal software-implemented shadow stacks may be vulnerable due to their inherent imperfections, as they can also become a part of the attack targets. To tackle these issues, we enhance the previous shadow stack by integrating a lightweight version into inaccessible hardware for the programmers. This new implementation only backs up valid return addresses during function calls and verifies actual return addresses at the returning stage. The depth of the lightweight shadow stack is determined by the maximum number of function iterations, which indicates the limit of the recursive calls. In the hardware implementation, the logic finite state machine can directly monitor the instructions and control signals in the processor pipeline, assert push and pop operations of return addresses at runtime, and perform backup and verification of the return addresses. The lightweight hardware shadow stack operates in parallel with the processor pipeline and remains transparent to the programmers.

To further enhance the security and integrity of crucial sensitive data associated with CFI, we propose a lightweight stream cipher circuit for runtime stack data encryption and decryption. This structure can dynamically encrypt and decrypt sensitive data in the stack. In this case, the dynamic stack data stored in external memory is encrypted, which prevents attackers from analyzing or tampering with forward-edge (indirect branch and calls) and backward-edge (returns) transfers, and ensures the security of program control flow. We then implement the hardware module that connects the processor D-Cache and system bus interface within the processor core. The cipher circuit is only activated when the D-cache is accessed in a missing status, effectively reducing the frequency of stack data encryption and decryption operations. Stream cipher circuits utilize the Advanced Encryption Standard (AES) in the counter model to encrypt program execution parameters, such as access address and access counters, instead of conventional data encryption. The encrypted execution parameters are then passed to XOR gates with the stack data to be encrypted, and the resulting output is stored in external memory as encrypted stack data. This enables parallel operation between cryptographic calculations and the program’s memory access. To achieve stack data encryption and decryption, it is sufficient to implement only the AES encryption hardware. This is because the encrypted stack data can be decrypted through an XOR operation with the encrypted execution parameters. The proposed lightweight stream cipher circuit is particularly suitable for embedded systems with limited hardware resources and performance overhead.

At last, we implement the proposed lightweight hardware shadow stack and the runtime data encryption hardware into the OR1200 processor, which was then mapped onto an Intel EP2C70 FPGA chip. We use a series of benchmark programs to evaluate both the security and performance overhead. The hardware overhead was assessed under SMIC 0.18μm 1P6M technology using Synopsys tools. The results indicate that the proposed hardware-assisted lightweight scheme can effectively verify the backward-edge transfers and resist malicious analysis of the crucial stack data, with less system impact of an additional 0.39% performance overhead and 0.316 mm^2^ area footprint.

The main contribution of this paper can be summarized as follows:

- A method for ensuring control flow integrity of backward edges based on the lightweight shadow stack is proposed. The hardware is integrated into the processor pipeline and collaborates with the instruction decoding module. In case the return address of any function does not match the valid parameter in the shadow stack, potential backward edges CRA attacks can be timely detected.
- A stream cipher-based lightweight runtime stack data encryption and decryption scheme for embedded systems is proposed. This scheme can block malicious analysis of the stack data that can be used to launch CRAs. This mechanism automatically encrypts stack data during the processor’s write-back phase and decrypts it when there is a cache miss at stack data loading, which is transparent for the programmers. The stream cipher circuits parallelize the process of memory access and parameter encryption, thereby reducing the system performance overhead. Additionally, the proposed scheme only requires to implement AES encryption hardware engine, resulting in considerable hardware savings.
- In addition, we integrated the aforementioned two mechanisms into the open source OR1200 processor and verified the system security function on the Terasic DE1-SoC FPGA board using a suite of selected benchmarks. The hardware overhead has been evaluated using Synopsys Design Compiler. The experimental results demonstrate that the proposed hardware-assisted lightweight scheme effectively protects embedded systems from code reuse attacks with minimal performance overhead and a small footprint.

The remainder of this paper is organized as follows. Section 2 presents the security threats to the embedded systems addressed in this work. Section 3 reviews the control flow integrity mechanisms. Section 4 demonstrates the design details of our proposed hardware-assisted lightweight scheme for ensuring control flow integrity from CRAs. Section 5 shows the experiment’s results and provides some discussions. Conclusions are drawn in Section 6.

## 2. Security Threats for This Work

Code reuse attacks can use existing instructions from valid libraries or executable files to construct an attack, without the requirement to inject malicious code into the vulnerable programs. At first, the adversaries conduct an analysis of libraries, executable files, and dynamic data in the dynamic memory. Then, they may build malicious gadget chains using valid instructions, such as branch and ret instructions, to create malicious program functions. During the subsequent running phase of the program, function pointers and return targets that control program flow may be tampered with to jump to these malicious gadget chains. Return-oriented Programming (ROP) and Jump-oriented Programming (JOP) are the primary methods for executing a code reuse attack.

### 2.1. ROP Attack

The ROP attack can be viewed as an advancement of the Return-to-Libc attack, where instead of invoking complete function code from the Libc library, it utilizes fragments of code that end with a ret instruction to achieve a Turing-complete code reuse attack. The attacker modifies the function’s return address and manipulates the program control flow to the prepared malicious gadget chains. Therefore, the ROP attack is closely related to the program call and return transfers.

**Figure 1:**
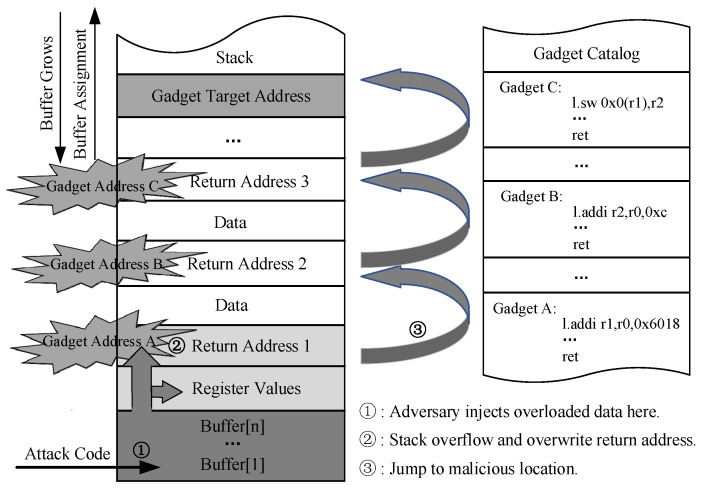
Conducting a ROP attack through buffer overflow.

Figure 1 presents a method to conduct a ROP attack through buffer overflow at runtime, which is one of the most prevalent methods to temper a function’s return address. Prior to execution, the attacker conducts a scan of existing binary files within the program to identify and capture gadgets capable of performing malicious operations. During program execution, when a function is called, a function stack frame is dynamically allocated in memory to pass parameters, save local variables, and store the return address. The attacker exploits buffer overflow vulnerabilities by injecting carefully calculated gadget addresses and related parameters into the stack, thereby overwriting the original function return address. Once the function returns, program execution may deviate from its intended path as it jumps to malicious gadget chains. The actual return address of the function serves as crucial metadata in detecting ROP attacks. Finally, the attacker continuously employs ret instructions to manipulate the execution sequence of gadgets and construct a complete malicious attack program, thereby hijacking the user’s program control flow to achieve the pre-defined attack.

### 2.2. JOP Attack

**Figure 2:**
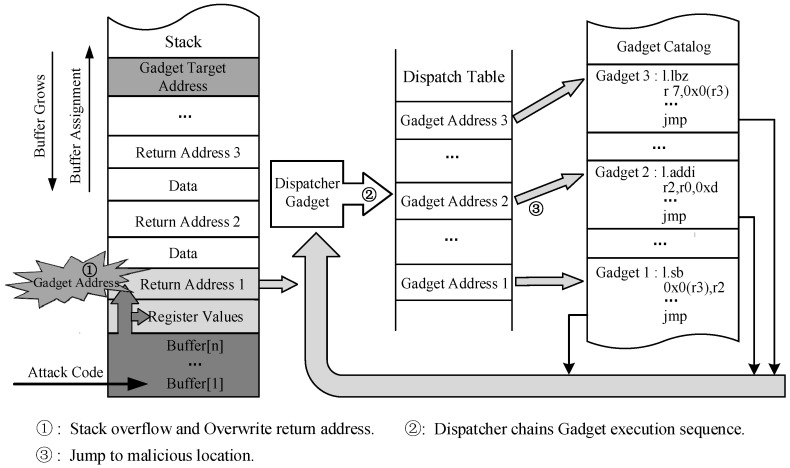
Conducting a JOP attack using dispatch table.

Similar to ROP attacks, JOP attacks also rely on existing gadgets to achieve specific functions and enable Turing-complete code reuse attacks. However, JOP attacks utilize indirect jump instructions instead of ret transfers to generate gadget chains. Therefore, the transfer of the malicious control flow depends on the destination address inferred from the registers used in the indirect jump instructions. The attacker collects the addresses and associated data of all possible gadgets to construct a dispatch table, which is then used to link gadgets by a malicious scheduler. Then, the target address stored in registers may be tampered with, leading to the execution of a JOP attack. A method to conduct a JOP attack using a dispatch table is demonstrated in Figure 2. Unlike ROP attacks that exploit backward edge transfers, JOP attacks primarily manipulate forward edge transfers to control the program execution flow.

We assume the regions within the embedded SoC chip are a trusted zone while regarding all interfaces and wires connected to the SoC, as well as all system components and peripherals off the chip, as untrusted. The indirect jump targets that are mapped to processor registers can only be maliciously analyzed and modified since they are stored off the chip. Therefore, dynamic stack data is a vulnerable point for conducting malicious attacks.

## 3. Related Works

In response to the security threat posed by code reuse attacks, extensive research has been conducted on defense methods against such attacks. These include CRA defense methods based on randomization [15,16], protection methods based on CFI protection [17], and Data Execution Protection (DEP) [18].

DEP adopts a protection strategy that prohibits programs from simultaneously writing to and executing the same memory section, in order to prevent attackers from executing malicious code injected by the attackers. However, this defense method is vulnerable to bypassing through ROP and JOP attacks [18]. Randomization techniques are an effective defense against CRA attacks as they randomize code segments, data segments, and control flow paths within a program. This makes it challenging for attackers to accurately predict the locations of code and data or the execution states of the program.

The ASLR mechanism employs a randomized selection process for the base addresses and sizes of code and data segments, allocating memory areas in accordance with pre-defined rules each time the program is executed [15]. This approach effectively thwarts attackers’ attempts to predict segment locations, thereby bolstering system security. Jin proposed a protection model called BoundShield, which utilizes the Software Fault Isolation (SFI) mechanism to establish an inaccessible confidential area, thereby preventing memory leakage attacks by concealing code segments and pointers [11]. However, Göktas devised a Position-independent Return-oriented Programming (PIROP) attack model that effectively circumvented the ASLR security mechanism without any information leakage, thereby demonstrating that code reuse attacks still pose a significant threat to ASLR [16]. It is especially prominent for bare-metal programs without operating system supports.

Instruction set randomization mechanisms [19] randomize the program’s instruction order, opcode, and register usage order according to pre-defined rules when the source code is compiled into binary executable files. Koo proposed a Compiler-assisted Code Randomization (CCR) mechanism in which the compiler analyzes the source code, generates an Abstract Syntax Tree (AST), and the rewriter performs code transformation and randomization based on the AST. However, this method entails transforming and randomizing a large amount of code in the program, leading to decreased code execution efficiency [20]. Wang investigated the impact of different randomization strategies on program security and performance, evaluating and analyzing several existing binary randomization techniques. Wang noted that the efficacy of randomization techniques varies depending on program characteristics, and there remains a risk of reused randomized code segments [12]. Existing randomization techniques have limitations in scope and effectiveness, often accompanied by significant performance overhead.

CFI protection mechanisms encompass various implementation methods, such as control flow validation and program and data encryption, which have proven to be one of the most effective methods in addressing CRAs.

### 3.1. CFI Protection Based on Control Flow Validation

Control flow validation methods directly monitor the transfer paths and critical parameters between program basic blocks and halt execution upon verification failure. Some of these techniques involve constructing a control flow graph (CFG) for the program through source code or binary code analysis [17,21]. The CFG is then utilized as a reference model to monitor runtime control flow transfers. Jung developed and implemented a vCFI defense mechanism that improves the effectiveness of CFI protection by employing static analysis of source programs, extracting control flow-related data, and monitoring and safeguarding runtime data vulnerable to control flow hijacking [1]. Nevertheless, the vCFI mechanism incurs a performance overhead as high as 13.6%. Barbar proposed a Live Path Control Flow Integrity (LPCFI) mechanism that implements forward-edge CFI by dynamically calculating CFG, thereby enhancing control flow detection accuracy and enabling the timely discovery of program control flow hijacking behavior [17]. Park introduced the BGCFI scheme, a fine-grained forward-edge CFI defense method based on bipartite graphs. The bipartite graph described the mapping relationships between all indirect branches and valid destination addresses. It was able to replace the verification of CFI with the edge presence problem in the bipartite graph when detecting whether the control flow transfer is legitimate [21].

The CFI protection method based on control flow validation prevents the attacker from arbitrarily calling code and data segments in memory. It allows them to construct attack programs and launch attacks using only a few legitimate target addresses, effectively protecting against code reuse attacks. The main drawback of these methods is that different programs have different CFGs. Whenever an updated version of a program is executed, the CFG must be regenerated. The size of the program and the inference target of the indirect jump/branch instructions both affect the construction process for CFG.

Some works focus on ensuring the parameters of program transfers, such as instruction sequences, basic block orders, function calls and returns, and stack data, which are critical information related to program control flow. Das designed a Basic Block CFI (BB-CFI), which extracts program control flow information and verifies the target address (TA) of control flow instructions that may be tampered with by attackers to monitor the legality of program execution flow [22]. Zieris proposed a Leak-Resilient Dual Stack Scheme, a backward-edge CFI defense method that uses stack separation technology to design a secure stack, storing program return addresses and related parameters to achieve information isolation and hiding, thus protecting system integrity [23]. This scheme relies on techniques such as the LLVM compiler, which can have some impact on system performance and compatibility. At the same time, the two-stack structure introduces significant hardware resource overhead. Zhang designed a dynamic shadow stack (RS-Stack) to save sensitive data, using shadow stack address randomization to prevent attackers from analyzing shadow stack locations. However, the performance overhead introduced by this shadow stack protection mechanism increased fivefold [6]. Lehniger combined label-checking technology with return address obfuscation technology, ensuring the legality of instruction calls through return address label checking, thereby preventing malicious attacks and illegal calls [24]. Oh introduced the Active Function List (AFL) for detecting control flow hijacking behavior. The detection algorithm enforces that return instructions can only return to active functions, and jump instructions must cross function boundaries. This algorithm determines whether it is susceptible to control flow hijacking through these function transfers [25].

### 3.2. CFI Protection Based on Program and Data Encryption

Aiming at the feature that CRA attacks need to reuse existing program sections, the CFI protection methods based on program and data encryption mainly encrypt sensitive data such as code blocks and control flow transfers. This encryption makes it difficult for attackers to obtain, analyze, and use these code segments, data segments, and control flow information to launch attacks and effectively reduces the threat of code reuse attacks. Encryption of sensitive data related to control flow information can be implemented from both software [26,27] and hardware [28,29] perspectives. Software implementations offer advantages such as strong flexibility. The downside is that the software implementation risks being reverse-engineered and bypassed, and the software itself can be vulnerable to attack and damage. Moreover, the software implementation introduces significant performance overhead for embedded systems. Hardware implementations can provide higher security, faster computation speed, and minimal impact on system performance, but at the cost of high implementation expenses and difficulties in updating and optimizing.

Software encryption implementation mainly involves obfuscation-based mechanisms [9,26] and dynamic encryption mechanisms based on encryption algorithms. Li designed an obfuscation algorithm for program control flow graphs. This algorithm transforms an inline function in a program into a contour function, hiding the basis-block jump relation in the process. Pseudo-functions and pseudo-control flows are also introduced to enhance obfuscation strength significantly, aiming to conceal the program’s control flow [9]. Suk designed an obfuscation tool called SCORE that provides control flow obfuscation techniques at the source code level, optimizing and refactoring programs while improving the readability of obfuscated source code [26]. While obfuscation techniques can effectively increase program security, imperfect obfuscation may introduce vulnerabilities and redundant code, affecting program performance and maintainability. Hiscock demonstrated a software encryption and hardware decryption scheme, which involves encrypting instruction-level code using a stream key before generating executable binary files and implementing code decryption by adding a hardware decryption module in MIPS soft-core [27]. However, decryption inevitably introduces a performance overhead that slows down the execution of the program.

Hardware-based program and data encryption methods incorporate hardware encryption and decryption modules within the system, encrypting program code and sensitive data through hardware and implementing dynamic decryption during program execution. To safeguard code and sensitive data in embedded processors, Savry proposed a memory encryption and authentication mechanism called CONFIDAENT, which primarily relies on instruction and data-authenticated encryption to enhance program execution confidentiality, preventing code reuse attacks and stack overflows [10]. Gueron designed a coprocessor to protect information security in memory. However, this approach lacks universality in resource-constrained embedded systems [28]. Zhang proposed a hardware-assisted control flow integrity checking mechanism to defend against code reuse attacks using the Hamming distance matching principle for encryption and linear encryption and decryption. This mechanism also uses dynamic key updating to enhance security [29]. Yang integrated the AES engine within a memory controller related to DMA and memory data transfer. The AES engine consumes extra hardware resources when multiple external memory devices are present in the system [30]. Wang proposed a scheme for encrypting code and storing data using the AES [31]. However, encryption with fixed-key introduces security risks such as key leakage. Encryption and decryption computations also significantly extend the memory access time. Moreover, the issue of additional hardware resources occupied by the cipher-computing module cannot be neglected.

## 4. Microarchitecture Design and Implementation

In this paper, we propose a lightweight hardware-assisted control flow integrity protection scheme to mitigate code reuse attacks. This mechanism primarily involves monitoring control flow information and encrypting critical sensitive data. We integrate a lightweight shadow stack into an inaccessible hardware isolation region, where the monitoring unit utilizes this shadow stack to examine transfer instructions and control information within the pipeline. This process validates the validation of backward edge target addresses in the program and promptly identifies any potential control-flow hijacking attacks. To further enhance the defense capabilities of embedded systems, we propose an efficient and secure structure for encrypting runtime stack data. By utilizing an optimized AES encryption engine working in counter mode and implementing a dynamic key update strategy based on critical parameters, we reinforce the confidentiality of sensitive data, thwarting attackers from analyzing both the forward-edge and backward-edge transfer processes of the program. By implementing these measures, we can proactively defend against CRA attacks.

### 4.1. Threat Model

**Figure 3:**
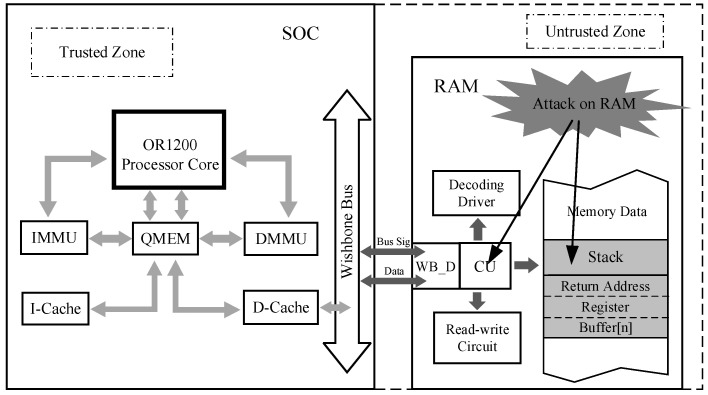
The threat model for this work.

To propose a security protection mechanism suitable for embedded systems, we first constructed a threat model and security assumptions based on common CRAs such as ROP and JOP attacks. We intend to incorporate the proposed security mechanism into the OR1200 processor, creating a processor SoC architecture that safeguards against code reuse attacks from a stack security perspective. The OR1200 is a 32-bit open source microprocessor with Harvard architecture, consist an on-chip DMMU and D-Cache. During program execution, CFI-related sensitive data such as function parameters, local variables, return addresses, and register data generated by function calls and jump operations are stored in the stack. Typically, the stack is located in external memory, which is vulnerable to malicious attacks. As all programs are executed under the control of the processor and implement specific functions, an attacker must hijack the program’s control flow to achieve their attack goals. This requires analyzing and exploiting the data in the stack. The processor, being a relatively enclosed component, is assumed to be secure and free from vulnerabilities or malicious programs in this work. In addition, we assume that malicious attacks originate from external sources and that the IP cores and other circuit units directly connected to the processor are most susceptible to such attacks. Therefore, securing stack data from external memory has a direct impact on system security. We consider this part of stack data as sensitive information related to CFI and protecting it is the main work of our security mechanism. The threat model depicted in Figure 3 illustrates that a malicious attacker has the capability to access and read data stored in external memory and external IPs at any time they will. Furthermore, they possess the ability to manipulate the data from the stack to construct malicious code chains to launch CRAs.

The following reasonable assumptions are made in this work: the internal area of the SoC chip is designated as a trusted zone, while the external area of the SoC chip is considered untrusted. Additionally, we define the stack area in off-chip memory as an untrusted critical region, and any sensitive data from this off-chip stack is deemed untrusted critical sensitive data. When program and stack data are stored in external memory, malicious attackers may exploit them to launch CRAs.

For embedded systems, there exist various malicious attacks that can cause different security problems. Establishing a certain security threat model can clearly identify the types of security threats faced by this paper’s research and highlight clear research issues to be addressed. It is obvious that some attacks can tamper with the data inside the SoC chip, but this is out of the scope of this paper. Therefore, under the security assumptions and threat model made by this paper, the work can focus only on off-chip data security issues. It should be noted that our security assumptions and threat model are reasonable. At present, the main way of security threats against embedded systems, especially for CRA attacks, is to intercept data stored outside the chip and break the integrity of forward and backward control flow transfers. This does not affect the overall validity of the threat model.

### 4.2. Microarchitecture Overview

**Figure 4:**
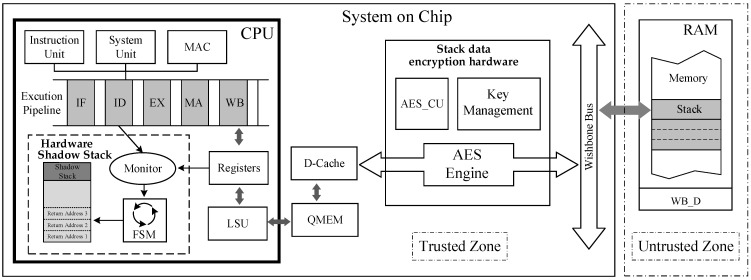
The Overview of the Proposed Mechanism.

By analyzing the mechanisms of ROP and JOP attacks, as well as the threat model, we have identified a critical component in the embedded SoC chip architecture that requires protection, namely, the stack unit located in off-chip memory. Subsequently, we designed and implemented a CFI security mechanism from the perspective of stack safety. This mechanism consists of a lightweight hardware shadow stack, which traces the validation of the backward-edge control flow transfers, and a runtime data encryption hardware concentrated on critical data encryption. The hardware overview of the proposed mechanism is presented in Figure 4.

### 4.3. Lightweight Hardware Shadow Stack

The detection mechanism employs a backward edge CFI method to implement control flow integrity, which enables the identification of CRAs during program execution. It is assumed that the attacker gains access and control over the external circuitry of the SoC chip in an attempt to manipulate the program’s control flow within the processor. According to the aforementioned analysis of the CRA attack mechanism, an attacker can obtain the instruction fragment capable of performing a specific operation from the existing code fragment and construct the program flow by calling a series of instruction fragments. Among them, ROP attacks exploit memory vulnerabilities to connect instruction fragments by manipulating the return address of ret instructions on the stack, thereby redirecting program execution flow to the desired fragment. It is evident that the attack involves tampering with the return address in the function stack frame, resulting in a misalignment of program breakpoints at function call and return. In the defense design, we incorporate a lightweight shadow stack into the hardware isolation area. This stack is dedicated to storing return addresses in function stack frames, ensuring that they cannot be accessed or tampered with by any program. To mitigate the risk of bypassing monitoring mechanisms and tampering with return addresses after popping from the stack, our CFI monitoring module directly monitors function calls and returns from the processor pipeline. At the function calling stage, the lightweight hardware shadow stack automatically stores the return address without backing up any other data from the original stack. Upon completion of execution and return to the calling function’s state on the stack, the system performs an automatic comparison and verification of the return address to ensure the validity of the program control flow. The CFI checking mechanism enables the detection of ROP attacks during each transmission, thereby indirectly verifying the security of sensitive data stored in the stack. Algorithm 1 shows a detailed description of the backward-edge control flow checking method.
**Algorithm 1:** The procedure of the backward-edge control flow checking**Input****:** Binary Instructions in Pipeline, PC, Shadow Stack, Parallel Decode**Output****:** *Security Status**EX_insn*← Pipeline instructions*W_addr*← Return address written into shadow stack during function call*R_addr*← Return address read from shadow stack during function return*EX_addr*← Address being executed in pipeline during function callST_addr← Return address obtained from memory during function return**For all** *EX_insn* **do**  *Opcode* = Parallel Decode (*EX_insn*)  *EX_addr* = *PC + 4* /* PC: Instruction address during the decoding phase */  **if** Opcode *= Function Call* **then**    *W_addr = EX_addr + 4*
    Shadow Stack ← *W_addr*  **else if** Opcode *= Function Return* **then**    *R_addr* ← Shadow Stack    while (*ST_addr* != *NULL*)     ;  /* Waiting for memory access to finish */    *Security Status* = (*ST_addr* = *R_addr*)? *NO_WARNING*: *WARNING*  **else**
    *Security Status* = *NULL***END**

**Figure 5:**
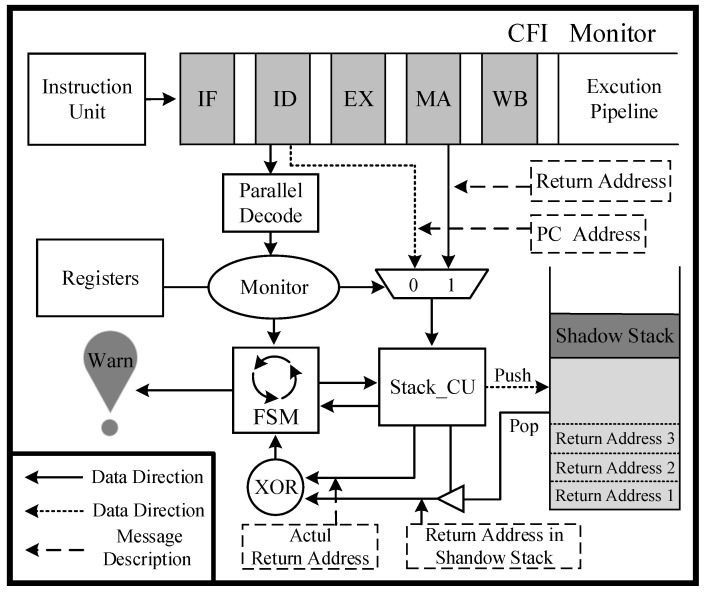
Hardware details of the lightweight hardware shadow stack.

Based on the analysis of the proposed lightweight hardware shadow stack, the hardware is implemented through three components: (a) monitoring unit, (b) lightweight shadow stack, and (c) control unit. The monitoring unit analyzes instructions executed in the pipeline at runtime through signals fetched from the pipeline, enabling it to monitor each function call and return. This allows for obtaining breakpoint addresses and return addresses during execution when functions are returned. The lightweight shadow stack is situated in a hardware-isolated enclave that is inaccessible to software and dedicated solely to storing return addresses within function stack frames. The control unit is implemented using a finite state machine, with backup and verification of the return address being completed based on feedback signals from the monitoring unit. The three components are implemented through hardware and operate automatically without the need for software control. The module’s contents can only be accessed by the control unit’s alarm signal, and the proposed module’s overall structure is illustrated in Figure 5.

The monitoring unit is dedicated to the command and control signals within the pipeline. The processor implements a five-stage pipeline comprising instruction fetch (IF), instruction decode (ID), execute (EX), memory access (MA), and write-back (WB). During the decoding phase of the pipeline, the monitoring unit acquires the binary code of instructions, jump signals, and program counters, verifying their correspondence with the function call and return signals. The OR1200 embedded processor is capable of supporting delay slot instructions, which means that it will not execute two jump instructions consecutively. In the event that a function call is detected by the monitoring unit, both the program counter read from the pipeline and the function call signal are sent to the control unit. Similarly, when a return instruction is detected, the monitoring unit retrieves the jump address from the rB register and transmits it to the control unit after the pipeline fetches the target address from the function stack frame. It should be noted that monitoring command and control signals in the pipeline does not alter its structure. Instead, it achieves this through the addition of a few minor components. Moreover, the proposed lightweight hardware shadow stack operates much faster than the instruction execution speed and is guaranteed not to impact pipeline performance.

Based on the features of a function call and return procedure, a lightweight hardware shadow stack is implemented in the trusted region of the SoC chip to store the return address of the function stack frame. The depth of the shadow stack depends on the maximum number of iterations of functions, which has an impact on both system security and hardware implementation cost. In the benchmarks we selected from MiBench embedded benchmark [32], the maximum function call iteration depth is in the quick sort test program, which is 16 layers. Therefore, we have set the shadow stack depth to 30 layers to provide redundancy. Depending on different application scenarios, the size of the shadow stack can be adjusted according to function iteration depth. Furthermore, this lightweight implementation only backups the return address, which does not impose any significant resource burden on embedded systems. To prevent malicious tampering of data in the shadow stack, hardware isolation measures are implemented in its design. This prohibits any program from accessing it and only allows the control unit to autonomously complete push and pop operations of return addresses, ensuring relatively high security of the shadow stack.

The control unit comprises a finite state machine that executes specific actions based on feedback signals received from the monitoring unit. Upon receiving a function call signal, the control unit enters backup mode wherein it automatically translates the program breakpoint pointer into the address at function return and pushes it onto the shadow stack. Upon receipt of a function return signal, the control unit enters the verification state wherein it performs a pop operation on the shadow stack and compares the return address stored therein with that executed in the pipeline to ascertain any potential tampering with the function return address. The verification method indirectly guarantees the security of data in the external stack of SoC chips. In case a malicious modification is detected in the return address, the control unit will enter an alarm state and send an alarm signal to the system. Users can write specific programs to protect their systems, such as halting all current work on the processor and running embedded security defense software in a secure memory region to further mitigate the risks of such attacks. Furthermore, in the event of a malicious attack causing an overflow of the shadow stack, the control unit will enter into an alarm state.

### 4.4. Runtime Data Encryption Hardware

The success of ROP and JOP attacks hinges on the availability of a chain of gadgets in the code. In order to invoke these gadgets, the program counter needs to be accurately redirected to the desired gadget. Attackers typically employ ret and jump instructions to achieve this program’s counter redirection. However, accurately redirecting the program counter from normal program execution to the gadget is a challenging task. To perform further data tampering, it is imperative to conduct a precise analysis of the stack data structure and its contents in order to locate relevant elements such as registers, return addresses, and available arrays that are crucial for program execution.

We consider information related to program execution and control flow transfers as critical and sensitive data. To hinder attackers from analyzing and identifying this data, we designate the stack data, mainly for the function stack frames, as sensitive data. By encrypting stack data with AES before storing it in external memory, even if attackers gain access to memory through high-privileged interfaces like JTAG and acquire read/write permissions for the data, they are unable to construct attack programs by analyzing and exploiting sensitive information. Therefore, a security mechanism that can encrypt stack data can provides defense against control flow-related CRAs.

To minimize the time overhead associated with stack data encryption and decryption processes, we have designed hardware for runtime stack data encryption, optimized the encryption structure, and implemented it internally within the SoC chip. This optimization ensures more efficient and secure encryption while mitigating the performance impact of encryption on stack data. This architecture enables fast encryption of sensitive data when it enters the stack during program execution, as well as efficient decryption when the data is fetched from the external memory. To enhance encryption efficiency, we have opted to integrate this encryption structure between the D-Cache and the system bus. D-cache’s working mechanism can effectively help to reduce the encryption and decryption frequency. The encryption module comprises two components: an encryption control unit and an AES encryption engine.

**Figure 6:**
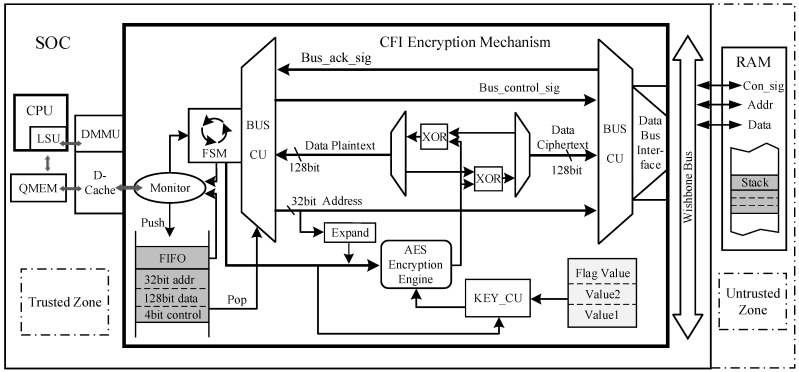
Hardware details of the runtime data encryption hardware.

Given the limited system resources of embedded systems, it is imperative to minimize the impact of encryption on both performance and hardware overheads when designing an encryption structure for such systems. Therefore, this paper integrates the AES encryption engine between the D-Cache and the system bus, designed within the trusted area of the SoC chip. As the stack units in the off-chip memory are located in the untrusted region, sensitive data must be encrypted when transferring from the trusted to the untrusted area to ensure data security. The initiation of this bus transfer is automatically controlled by the hardware BUS CU and, in conjunction with the encryption key and XOR circuit, achieves encrypted storage of sensitive data and decryption during data access. The monitoring circuit in the encryption control unit is responsible for checking the D-Cache hit status and data transfer direction, providing feedback signals to the FSM within the control unit. Sensitive data is integrated into data blocks under the control of FSM, which are then prepared for encryption and stored in the encryption FIFO along with bus signals. The KEY_CU manages the critical information, generating non-reproducible encryption keys based on feature values against CRA attacks and the accessed address information. Figure 6 illustrates the encryption architecture for stack data. It is important to note that in this design, the content encrypted using the AES is the sensitive data, and the process involves XOR operations between the encrypted key and plaintext or cipher text. The AES engine is responsible for encrypting the encryption key itself.

The encryption mechanism for sensitive data in the stack operates as follows: When the processor accesses sensitive data, it first accesses the trusted region’s D-Cache. If there is a hit in the D-Cache, the processor directly performs read or modification operations on the data. In the case of a D-Cache miss, the cache initiates the update strategy to replace the existing cache block. During the cache block update process, the D-Cache establishes a data path with the stack units located in the external memory through the system bus, enabling burst transfers of 16-byte cache blocks between the cache and the stack units. However, it is important to note that the stack, which holds the sensitive data, resides in the untrusted region. To address this, an encryption control unit is introduced during the data transfer process. Sensitive data written to the stack units through the system bus undergoes encryption using AES. Similarly, sensitive data read from the stack units undergo AES decryption. The AES encryption operates on 128-bit data, matching the block size in the update strategy. Leveraging the high hit rate of the D-Cache, the AES encryption engine is only activated when a cache miss occurs, significantly reducing the frequency of encryption and decryption operations and minimizing their performance impact on the system. Moreover, the bus transfer process for this segment is controlled by the encryption unit hardware, effectively preventing external malicious attacks from circumventing the encryption unit to obtain unencrypted data.

The encryption control unit comprises a finite-state machine with four primary operational states: monitoring, missing, encryption, and decryption.

(1) In the monitoring state, the state machine assesses the hit status of the D-Cache primarily based on the cache_miss signal within the DC_FSM. If there is a hit in the D-Cache upon accessing, the state machine remains in the monitoring state. Otherwise, it transitions to the missing state.
(2) In the missing state, the encryption control unit obtains a 128-bit encryption key and feeds it into the AES encryption engine. If the D-Cache data needs to be written back to the stack units, the state machine transitions to the encryption state. If the D-Cache needs to access data in the stack units, the state machine enters the decryption state.
(3) In the encryption state, the encryption control unit stores the encrypted data in the 164-bit encryption buffer, which includes 128 bits of plaintext, a 32-bit storage address, and a 4-bit bus control signal. The plaintext represents the stack data to be encrypted, while the cipher text represents the encrypted stack data. It is important to note that once the encrypted information is obtained, the AES encryption engine immediately performs the encryption using the encryption key. Simultaneously, a response signal is sent to the processor to initiate the data write-back process, preventing the processor from remaining in a stalled state for an extended period. From the perspective of the processor, the duration of an encryption storage operation is equivalent to that of a regular data block storage. Upon completion of the key encryption, the plaintext is encrypted by applying the XOR operation between the plaintext and the encrypted key. Subsequently, the BUS CU stores the resulting cipher text into the stack unit based on the information stored in the encryption buffer.
(4) In the decryption state, once the encryption control unit provides the encryption key and initiates the start signal, the AES encryption engine immediately enters the operational state. Unlike the previous decryption method where the cipher text had to wait for memory access to complete before decryption, causing a delay in the processor’s data retrieval and leading to performance loss, the optimized encryption/decryption architecture introduces parallel processing. In the optimized encryption/decryption architecture, the bus transfer for access operations starts after waiting for the bus to become idle. Meanwhile, the AES encryption engine is already activated before the bus transfer, allowing the decryption computation and memory access processes to run in parallel. After completing the key encryption and obtaining the encrypted data blocks from the stack, the final decryption step is performed through the XOR operation between the encryption key and the encrypted data blocks. The duration of one decryption access operation depends on the maximum duration of memory access and key encryption computation. This effectively reduces the processor’s waiting time and improves the efficiency of encryption and decryption operations.

**Figure 7:**
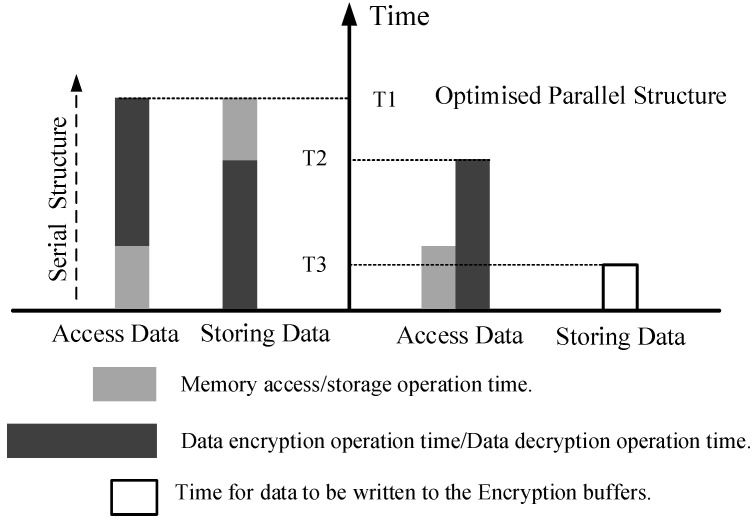
Comparison of memory operation time after encryption structure optimization.

Figure 7 provides a visual representation of the access operation time for different encryption methods.

The data paths for both AES encryption and decryption blocks are separated and essentially self-contained, requiring access to a set of round keys and a data block [33]. This design approach leads to a significant reduction of approximately 50% in the circuit area of the AES core when only the encryption circuit is implemented, after trimming the decryption circuit portion. Considering the constrained hardware resources of embedded systems, we opted to construct the hardware-based section of the 128-bit AES encryption circuit to minimize the overall circuit area of the AES engine.

The encryption of sensitive data in this article utilizes a stream cipher encryption method by AES counter mode. The encryption key plays a crucial role in the security of the encryption algorithm and is considered the most sensitive component. Traditional fixed-key encryption methods present security risks due to potential key leakage. Additionally, attackers may attempt to deduce the encryption key by analyzing the plaintext and cipher text of multiple encrypted datasets. To mitigate these risks and enhance security, the key used in the XOR operation requires a dynamic update mechanism, and it is encrypted by the AES engine.

This paper introduces a dynamic key update mechanism based on specific characteristics, referred to as “flag value”, to ensure security. The key to be encrypted consists of two parts: the storage address of the sensitive data and the CRA attack flag values. It is essential to note that the AES engine does not directly store this key. Instead, it retains the CRA attack flag values. To enable the AES encryption engine to promptly obtain the key to be encrypted before the state machine enters the encryption and decryption state, the transformation relationship between the key and the flag values is shown in Algorithm 2.
**Algorithm 2:** Initial key generation process**Input:** Access address for the stack, number of accesses to the stack address**Output:** *Key**Flag* ← Number of accesses to the stack address*Key_addr* ← Access address for the stack**For all** *Key_addr* **do**  *tempH* ← Expend(*Key_addr*) [127:64]  *tempL* ← Expend(*Key_addr*) [63:0]   While (*Flag*>0)    *Swap [16/8/4/2/1: 0]* = *tempH [16/8/4/2/1: 0]*     *tempH* = *tempH* and *tempL [16/8/4/2/1: 0]*    *tempL* = *tempL* and *Swap [16/8/4/2/1: 0]*    *Flag* = *Flag* − *16/8/4/2/1*  *Key* = {*tempH, tempL*}**END**

During the analysis of the CRA attack and the defense mechanism, it has been observed that the CRA attack relies on accessing sensitive data stored in the stack, necessitating frequent stack accesses. To effectively mitigate such attacks, we utilize the access frequency of sensitive data block addresses during the D-Cache update process as flag values. By leveraging this approach, each time-sensitive data is accessed, the encryption key undergoes modifications based on the corresponding access address and its associated flag values. This dynamic key update mechanism significantly enhances the security of the encryption algorithm, ensuring robust confidentiality during both encryption and decryption operations. The key initial and update method involves swapping the high and low bits of the data block address, with the number of bits exchanged determined by the flag values. The swapped bits directly form the initial key, ensuring timely access for the AES encryption engine while maintaining symmetry between the encryption and decryption keys. This dynamic key update mechanism helps to enhance the security of AES, ensuring robust confidentiality during both encryption and decryption operations.

Furthermore, the encryption control unit automatically records and manages flag values to enhance confidentiality and security. The system design employs hardware isolation techniques to strictly prohibit unauthorized access or manipulation of flag values by software components, ensuring the integrity and security of the encryption system.

## 5. Experiments and Discussions

We integrated the proposed lightweight shadow stack and stack data encryption hardware into an OR1200 embedded processor, constructing a SoC prototyping capable of against CRAs. The main frequency of the SoC is configured at 50 MHz, and the clock frequency of the proposed hardware is synchronized with the SoC. The internal D-Cache of the processor is configured to be 8 KB in size, with support for other sizes such as 4 KB and 16 KB. We utilized Verilog HDL to develop the system, conducted logic synthesis and implementation in Quartus Prime 18.1, and subsequently mapped the hardware implementation onto the Intel EP2C70 FPGA chip.

### 5.1. Hardware Overhead

The hardware resources of embedded systems are severely constrained. In light of practical considerations, we must ensure that the designed hardware security mechanism will not cause too much overhead to the hardware resources of the system. Our security mechanism is implemented on-chip and the overhead of hardware resources is more important.

We have completed the FPGA verification of the entire system on the Terasic DE1-SoC FPGA development board and evaluated its hardware overhead both using Quartus Prime and Synopsys tools under SMIC 0.18 μm 1P6M technology. During implementation, the lightweight shadow stack and stack data encryption engine were found to consume most additional hardware resources. We aim to optimize the hardware overhead in both architecture design and implementation, ensuring that the proposed hardware scheme is suitable for a wide range of embedded systems.

The depth of the shadow stack is related to the maximum number of iterations of the function, and its depth affects both the security of the system and the cost of hardware implementation. Insufficient depth of the shadow stack can result in inadequate handling of the function call depth by the backward-edge control flow validation, while excessive depth increases the hardware resource overhead of the embedded system. After analyzing the embedded benchmarks selected from Mibench, we found that the required shadow stack depth is between 9 and 15 layers, as shown in Table 1.

In order to provide redundancy, we decided to set the shadow stack depth supporting to 30 layers, with a storage size of 120 bytes. The hardware resource overhead for the shadow stack memory to store return addresses is 385 Logic Cells and 773 Registers for the FPGA end, as shown in Table 2. In different application scenarios, the shadow stack size can be expanded.

In the process of designing an AES hardware engine, there are two ways to implement the encryption and decryption hardware. One is to directly utilize the AES encryption circuit for data encryption and the decryption circuit for data decryption. However, this approach requires both circuits to be included in the hardware implementation, which increases the hardware resource overhead of embedded systems. Another approach, namely the stream cipher encryption method or AES counter mode method, relies solely on the AES encryption circuit for executing encryption operations to generate a PAD, and then the PAD is used to XOR with the data to be encrypted and decrypted.

We used the stream cipher encryption method for sensitive data encryption and decryption operations during the AES encryption engine design process. Therefore, we trimmed the AES decryption circuit and only retained the encryption circuit part to reduce hardware resource overhead. The overhead resource comparison of the AES engine after trimming the decryption circuit part is shown in Table 3. The hardware resource overhead is evaluated both target to the FPGA platform and ASIC.

Finally, we evaluated the hardware resource overhead from the complete lightweight hardware-based CRA defense mechanism. Table 4 describes the hardware resource usage for both the FPGA platform and ASIC. The results show that this defense mechanism brings a relatively small resource overhead to embedded systems, improving the practicality of the design.

### 5.2. Performance Overhead

Another crucial aspect of embedded systems is their high real-time requirements and limited system performance. The design process should minimize the impact on embedded system performance. This section evaluates the performance overhead caused by the CRA defense mechanism. The lightweight shadow stack is implemented by adding components to the pipeline while preserving its original structure. Moreover, the hardware-based checking logic executes significantly faster than the pipeline itself. Therefore, the impact of the lightweight shadow stack on system performance is almost negligible. However, the encryption-based CFI protection mechanism inevitably introduces performance overhead to embedded systems during the encryption and decryption of sensitive data. This paper primarily assesses the performance overhead caused by the encryption-based CFI protection mechanism.

During the encryption process of sensitive data, we effectively reduced the frequency of encryption and decryption operations by taking advantage of D-Cache, minimizing the impact of cipher operations on system performance. It is important to note that since malicious attacks originate from outside of the SoC, reducing the frequency of encryption and decryption does not compromise the security of the encryption effect. Concurrently, the design of the encryption cache area, parallel processing of decryption key calculation and memory access operations, and the fast XOR encryption and decryption method effectively reduce the time for individual encryption and decryption operations.

In the performance evaluation, we used program runtime as the performance metric. We selected ten different benchmark programs from the MiBench suite to evaluate the performance of the stack data encryption and decryption hardware. Table 5 illustrates the performance overhead introduced by the proposed CRA defense mechanism with the OR1200 D-Cache in size of 8KB.

In most benchmarks, the performance overhead caused by the defense mechanism does not exceed 2.68%, with an average performance overhead of 0.39%. The performance overhead of the OpenECC program is significantly higher than the average value because the encryption mechanism for sensitive data mainly works when accessing a D-Cache miss. During the cache block update process, the defense mechanism introduces additional clock cycles. The lower the D-Cache hit rate of the test program, the higher the frequency of AES engine encryption and decryption, and the greater the performance overhead caused by the defense mechanism. To this end, we configured the size of the D-Cache to 4 KB, 8 KB, and 16 KB, respectively, and used these 10 benchmark test programs to evaluate the performance overhead of the defense mechanism under different D-Caches. The experimental results are shown in Table 6.

The results show that the system’s performance overhead is related to the hit rate of D-Cache. The larger the D-Cache, the higher the hit rate, the lower the frequency of AES engine encryption and decryption, and the smaller the system’s performance overhead. The impact of the defense mechanism on embedded systems is weaker. In particular, the two groups of programs, OpenECC and blowfish, increase with D-Cache, and the performance overhead from the defense system decreases significantly.

### 5.3. Security Analysis

In the lightweight shadow stack validating the backward-edge control flow transfers, the monitoring unit directly monitors the function call and return processes from the pipeline perspective, preventing CRA attacks from bypassing the checking mechanism. During the function call process, the hardware shadow stack automatically backs up the return address. Upon completion of the function and returning the parent function state from the stack, the monitoring unit validates the backward edge transfer by verifying the return address, indirectly ensuring sensitive data security within the stack. If an illegal location is targeted for transfer, the checking mechanism will raise an alert, thwarting further execution of malicious programs. In designing the shadow stack, we implemented hardware on-chip and prevented access by any program to ensure that it would not be tampered with by malicious attacks.

In the encryption-based CFI protection mechanism, to prevent attackers from analyzing and locating critical sensitive data related to CFI, we classify all stack data where the function stack frames are located as sensitive data and provide encryption protection for it. Thus, the stack data stored in external memory cannot be directly analyzed and manipulated by adversaries. This ensures the security of the program during both forward-edge and backward-edge transfers. The encryption process employs a stream cipher encryption method to encrypt sensitive data rapidly. The security of the encryption is linked to the security of the cipher key. We implemented a dynamic key update mechanism by transforming the addresses of sensitive data based on the feature values of CRA attacks. This guarantees the security of the sensitive data encryption effect, making it hard for the attackers to locate and analyze sensitive data even if they have obtained read and write permissions for the stack data.

We compare the proposed CRA defense mechanism with some other previous works, as shown in Table 7. This article categorizes defense mechanisms into two security levels:

- Level I: can only defend against ROP attacks;
- Level II: can defend against both ROP and JOP attacks.

Compared to the software CFI checking mechanism, the hardware-implemented CRA defense mechanism achieves pipeline signals instruction-level monitoring, making it difficult for malicious attack programs to bypass and effectively detect control flow tampering behavior. Moreover, the implementation presented in this paper exerts minimal impact on the performance of embedded systems, with a performance overhead below 2.68% and an average of 0.39%. A stack data encryption circuit significantly enhances encryption and decryption speeds compared to software encryption. Compared to fixed-key encryption methods, the dynamic key update mechanism makes it difficult for attackers to infer the key even if they have obtained the plaintext and cipher text.

## 6. Conclusions

Considering the inherent features of embedded systems, such as stringent real-time requirements, limited performance capabilities, and prohibitive additional hardware overheads, this paper proposes a lightweight hardware-assisted security scheme to address CRAs in embedded systems. This mechanism is implemented with a focus on stack data security, primarily involving backward-edge control flow validation and runtime CFI protection through sensitive data encryption. (1) The CFI verification employs a method for ensuring control flow integrity of backward edges based on the lightweight shadow stack. It verifies the match between the return address of any function and the valid parameter in the shadow stack, enabling timely detection of potential backward edges CRA attacks. (2) CFI protection employs a stream cipher-based lightweight runtime stack data encryption and decryption scheme, which dynamically encrypts and decrypts stack data at runtime using the AES counter mode. This approach significantly enhances the security and integrity of crucial sensitive data associated with CFI, effectively preventing malicious analysis of the stack data that could be used to launch CRAs.

In the implementation, we have taken into full consideration the features of embedded systems and employed various techniques to minimize hardware resource overhead while reducing the impact on system performance. Finally, we integrated both mechanisms into the OR1200 processor. We verified the system security function on the DE1-SoC FPGA platform and evaluated the hardware overhead using the Synopsys Design Compiler. The results indicate that the proposed lightweight hardware-assisted scheme can provide a dedicated defense capability against code reuse attacks for embedded systems, with a hardware resource overhead of only 0.316 mm^2^ and an average system performance overhead of 0.39%.

## Figures and Tables

**Table 1:** Max. iterations and shadow stack depths for the selected benchmarks.

Benchmark	Functions Num.	Max. Iterations	Shadow Stack Depth
AES	57	10	9
OpenECC	81	10	9
Quicksort	47	16	15
bitcount	60	10	9
blowfish	47	10	9
patricia	49	15	14
SHA1	53	11	10
FFT	78	13	12
CRC	46	10	9
basicmath	70	11	10

**Table 2:** FPGA resource used for the proposed lightweight shadow stack.

Resource Utilization	Logic Cells	Registers	Memory Bits
Shadow Stack Memory	385	773	0
Whole Shadow Stack	488	781	0

**Table 3:** Comparison of hardware resource overhead of different AES engine implementations.

Resource Utilization		Full AES	Encryption-Only AES	Saves
FPGA	Logic Cells	2243	1118	50.2%
	Registers	1011	388	61.6%
	Memory Bits	1408	0	-
ASIC	Total Cells	26,045	12,168	53.3%
	Total Area	0.551 mm^2^	0.227 mm^2^	58.8%

**Table 4:** Hardware implementation overhead of the proposed system on FPGA and ASIC.

Resource Utilization		Original	Security Enhanced	Overhead
FPGA	Logic Cells	5426	7106	31.0%
	Registers	5284	5930	12.2%
	Memory Bits	416,492	416,768	0.1%
ASIC	Total Area	5.496 mm^2^	5.812 mm^2^	5.7%
	Power Consumption	83.17 mW	92.43 mW	11.1%

**Table 5:** Performance overhead of the proposed CRA defense mechanism with 8KB D-Cache.

MiBench	Total Insn	Original		Security Enhanced		Performance Overhead
		Runtime (Clock)	Insn Time	Runtime (Clock)	Insn Time	
AES	289,938	1,004,453	3.464	1,008,833	3.479	0.436%
bitcount	1,161,921	2,908,610	2.503	2,922,885	2.516	0.491%
basicmath	2,214,608,140	6,419,201,007	2.899	6,434,932,285	2.906	0.245%
blowfish	933,371	3,313,098	3.550	3,337,085	3.575	0.724%
CRC	295,164	772,774	2.618	777,768	2.635	0.646%
FFT	779,207	2,314,083	2.970	2,322,604	2.981	0.368%
OpenECC	122,316,893	406,662,908	3.325	417,553,731	3.414	2.678%
patricia	5,171,456	12,891,252	2.493	12,911,580	2.497	0.158%
quicksort	1,810,410	4,832,696	2.669	4,836,078	2.671	0.070%
SHA1	625,490	1,796,576	2.872	1,810,651	2.895	0.783%
Total	2,347,991,990	68,55,697,457	-	6,882,413,500	-	-
Average	-	-	2.920	-	2.931	0.390%

**Table 6:** Performance overhead of the benchmarks with the different sizes of D-Cache.

MiBench	4 KB D-Cache	8 KB D-Cahe	16 KB D-Cache
AES	0.457%	0.436%	0.434%
OpenECC	2.691%	2.678%	0.005%
quicksort	0.070%	0.070%	0.070%
bitcount	0.846%	0.491%	0.185%
blowfish	4.974%	0.724%	0.720%
patricia	0.575%	0.158%	0.092%
SHA1	0.880%	0.783%	0.363%
FFT	0.715%	0.368%	0.368%
CRC	0.840%	0.646%	0.605%
basicmath	0.272%	0.245%	0.245%

**Table 7:** Comparison of our work with some other works.

Security Mechanism	Security				Implementation			
	Level	Inst-Level Protection	Encryption Data	Key Risk	ISA Extensions	Modify Compiler	Hardware Overhead	Performance Overhead
vCFI [1]	Ⅰ	Yes	No	-	No	Yes	Medi	Medi (13.6%)
CFI [10]	Ⅱ	Yes	Yes	Medi	Yes	Yes	Low	High (36.0%)
LEA-AES [13]	Ⅱ	Yes	Yes	Medi	No	Yes	Low	Low (3.19%)
Dual Stack [23]	Ⅰ	Yes	No	-	Yes	Yes	High	Low (2.72%)
LLVM-LSE [27]	Ⅰ	Yes	Yes	High	No	Yes	High	High (48.0%)
HCIC [29]	Ⅱ	Yes	Yes	Low	No	No	Medi	Low (0.95%)
EC-CFI [34]	Ⅱ	Yes	Yes	Low	No	Yes	High	High (27.0%)
**Our work**	**Ⅱ**	**Yes**	**Yes**	**Low**	**No**	**No**	**Low**	**Low (2.68%)**

## Data Availability

The data presented in this study are available on request from the corresponding author.

## Abbreviations

The following abbreviations are used in this manuscript:

AES	Advanced Encryption Standard
AFL	Active Function List
ASLR	Address Space Layout Randomization
AST	Abstract Syntax Tree
BB-CFI	Basic Block CFI
CCR	Compiler-assisted Code Randomization
CFG	Control-flow Graph
CFI	Control-flow Integrity
CRA	Code Reuse Attack
DEP	Data Execution Protection
ICT	Indirect Control-flow Transfer
JOP	Jump-oriented Programming
LEA-AES	Lightweight Encryption Architecture based on Advanced Encryption Standard
LPCFI	Live Path Control Flow Integrity
PIROP	Position-independent Return-oriented Programming
RCFI	Random Control Flow Integrity
ROP	Return-oriented Programming
SFI	Software Fault Isolation
SOFIA	Software and Control Flow Integrity Architecture
TA	Target Address
UCT	Unique Code Target
vCFI	Value-based Constraint Control Flow Integrity
W^X	Write XOR eXecution
XOR	eXclusive OR
